# Distal capitate shortening with capitometacarpal fusion for management of the early stages of Kienböck’s disease with neutral ulnar variance: case series

**DOI:** 10.1186/s13018-014-0086-3

**Published:** 2014-10-11

**Authors:** Ezzat H Fouly, Ahmed F Sadek, Mohammed F Amin

**Affiliations:** Orthopaedic Surgery Department, El Minia University Hospital, Minya, Egypt; Radiology Department, El Minia University Hospital, Minya, Egypt

**Keywords:** Kienböck’s disease, Capitate shortening, Capitometacarpal fusion, Neutral ulnar variance, Scapho-capitate angle, Miniplate

## Abstract

**Background:**

The aim of surgical management of Kienböck’s disease has been proposed to slow the progressive osteonecrosis and secondary carpal damage. The aim of this case series was to evaluate the results of a new technique, combining distal capitate shortening with capitometacarpal fusion for the treatment of Kienböck’s disease (Lichtman stage II or stage IIIA) in neutral ulnar variance patients.

**Methods:**

From 2009 to 2012, 12 patients (mean age: 25 ± 7.6 years) were enrolled in this series. Radiological and clinical evaluations using the modified Mayo wrist scoring system were performed both pre-operatively and 12 months post-operatively. In addition, values of the scapho-capitate angle were evaluated both pre-operatively and 12 months post-operatively. The mean follow-up was 20.7 ± 11.2 months. Statistical analysis was performed for comparisons between pre-operative and post-operative findings with the use of paired sample *T* test, Pearson’s correlation, independent sample *T* test, and Spearman’s rho correlation. Statistical significance was determined to be present at *p* <0.05.

**Results:**

All patients achieved bony union at the fusion site within a mean period of 11.5 ± 2.4 weeks. Regarding wrist pain, grip strength, total wrist arc of motion, practicing daily activities in a normal pattern, and the total modified Mayo wrist score, there were statistically significant differences between the pre-operative and post-operative results. For the differential arc of motion, the only non-significant results were at the ulnar/radial deviation range (*p* = 0.262). The mean pre-operative scapho-capitate angle was 29.75 ± 3.44 while the mean post-operative value was 33.67 ± 4.77 (*p* < 0.001). Both pre-operative and post-operative scapho-capitate angle values were positively correlated to post-operative pain, ulnar/radial deviation, and final score (*p* = 0.001, 0.027, 0.021 and *p* = 0.001, 0.004, 0.002, respectively). Other parameters had no correlation to this angle.

Post-operative MRI (at 12 months follow-up) demonstrated better lunate revascularization in four patients; one of them was diagnosed as having Lichtman stage IIIA Kienböck’s disease. There were no patient-reported complications at the end of follow-up.

**Conclusions:**

Distal capitate shortening combined with capitometacarpal fusion represents a new reliable method in the treatment of early stages of Kienböck’s disease with neutral ulnar variance.

## Background

Kienböck’s disease is an osteonecrosis of the lunate; however, its precise etiology is unknown [1]. That is why its treatment is still empirical. The aim of surgical management has been proposed to slow the progression of osteonecrosis and secondary carpal damage or collapse. Factors defining the proposed treatment include severity of lunate damage, carpal stability, and presence of degenerative changes [1,2]. Some authors believe that as long as there is incomplete collapse of the lunate, fixed scaphoid rotation and only minor cartilage damage, disease progression and carpal malalignment might benefit from relief of load on the lunate [2].

In neutral ulnar variance, representing almost 8% of Kienböck’s cases, capitate shortening with or without capitohamate (CH) fusion has been proposed with promising results [3,4]. The aim of such procedure was of double fold benefit; lunate preservation by relieving imposed load and improving vascularization [2]. Although capitate shortening for Kienböck’s disease decompresses the radiolunate joint, the distal carpal row exhibits post-operative proximal migration with subsequent carpal malalignment. Capitate shortening combined with capitohamate fusion (CSCHF) was developed by Almquist and provided, in the authors view, more decompression of the radiolunate joint than leveling procedures such as radial shortening or ulnar lengthening [5]. However, some other authors reported that carpal collapse is ensued in all patients treated with CSCHF and moderate limitation of range of motion remained; although, pain relief and increased grip strength were noted [6]. After CSCHF, the scaphoid progressively adopts an abnormal palmar flexed position, followed by proximal migration of the distal carpal row until capitolunate contact is reestablished [6]. We believe that structural integrity of the scapho-capitate joint is the key for maintaining carpal alignment. In addition, the 3rd metacarpal is the most fixed and less mobile metacarpal, affording more stability in case its base is fused with the distal part of the capitate. Therefore, to prevent the proximal migration of the distal carpal row, we modified CSCHF and developed a distal capitate shortening procedure, in which the distal end of the capitate with the base of the 3rd metacarpal were denuded from their articular cartilage with capitometacarpal fusion.

The aim of this case series was to evaluate the results of this new technique, paving the way for further future assessment of its biomechanical validity.

### Patients and methods

Twelve patients who were primarily diagnosed with early Kienböck’s disease and were operated at our university hospital in the period from January 2009 till June 2012 were analyzed. The ethical committee review board of El Minia Faculty of Medicine approved this study on December 2008. A full informed consent was obtained from each patient enrolled in this study. They were five women and seven men (mean age; 25 ± 7.6 years, range; 18–41 years) (Table 1). All of the patients were right handed with symptomatic eight right wrists and four left wrists. Nine patients were manual workers. All patients complained of wrist pain, weak grip, and/or limited range of motion. The modified Mayo wrist score [7] was used to evaluate the patients’ clinical condition both pre-operatively and post-operatively (Table 2). This scoring system depends on evaluation of four parameters; pain, range of motion in percentage to the sound side, grip strength percentage to the sound side, and the functional status. Each parameter was evaluated and given a score of points; 0, 15, 20, or 25 and the sum of the points given for all parameters represented the final score for each patient that was subsequently interpreted into grades; excellent, good, satisfactory, or poor. Grip strength was measured with a Jamar Hand Dynamometer (expressed as percentage of healthy side strength). The pain scale was self-reported and graded according to the questionnaire provided by the modified Mayo wrist score. The total and differential arcs of motion of the wrist (extension/flexion, ulnar/radial deviation, and pronation/supination) were measured using a two-arm goniometer (expressed in percentage of healthy side). The patients’ pre-operative score averaged 57.9 ± 8.4 points (range; 45–70 points) (Table 3).

**Table 1:** **Patient demographics**

**Case no.**	**Age (years)**	**Side**	**Gender**	**Lichtman staging**	**Fusion healing time (weeks)**	**Follow-up (months)**
*1*	19	L	M	II	8	12
*2*	20	R	F	IIIA	10	24
*3*	18	R	M	IIIA	9	15
*4*	38	L	F	II	12	27
*5*	26	R	M	IIIA	11	47
*6*	20	R	F	II	10	13
*7*	21	L	M	II	11	20
*8*	22	R	M	II	14	12
*9*	25	R	F	II	12	12
*10*	24	R	M	IIIA	10	36
*11*	41	L	M	II	16	18
*12*	32	R	F	II	15	12
*Mean ± SD*	25 ± 7.6				11.5 ± 2.4	20.7 ± 11.2

**Table 2:** **Modified Mayo wrist scoring** [7]

	**Description**	**Score**
*Pain intensity*	No pain	25
	Mild occasional	20
	Moderate, tolerable	15
	Severe to intolerable	0
*Functional status*	Return without protection	25
	Return with protection	20
	Restricted return to work	15
	Unable to return to work	0
*Range of motion (% of normal side)*	90%–100% (normal)	25
	80%–89%	20
	70%–79%	15
	50%–69%	0
*Grip strength (% of normal)*	90%–100% (normal)	25
	80%–89%	20
	70%–79%	15
	50%–69%	0
*Total score interpretation*	*Excellent (90–100), good (80–90), atisfactory (60–80), and poor <60.*

**Table 3:** **Results of the patients both pre and postoperatively**

**Patient (N)**	**Pain**		**Functional status**		**Range of motion**		**Grip strength**		**Total score**		**Score interpretation**
	**Pre-**	**Post-**	**Pre-**	**Post-**	**Pre-**	**Post-**	**Pre-**	**Post-**	**Pre-**	**Post-**	
*1*	15	25	15	25	20	20	15	25	65	95	E
*2*	15	20	15	20	15	20	15	25	60	85	G
*3*	0	20	15	20	15	20	15	20	45	80	G
*4*	0	20	15	25	15	20	15	20	45	85	G
*5*	0	20	15	25	15	20	15	15	45	80	G
*6*	15	25	15	20	15	20	15	25	60	90	E
*7*	15	20	15	25	20	20	20	20	70	85	G
*8*	15	20	15	20	15	20	15	20	60	80	G
*9*	15	20	15	25	15	20	15	25	60	90	E
*10*	15	25	15	25	15	20	15	25	60	95	E
*11*	15	25	15	25	15	20	15	15	60	85	G
*12*	15	25	15	25	20	25	15	25	65	100	E
*Mean ± SD*	11.3 ± 6.8	22.1 ± 2.6	15	23.3 ± 2.5	16.3 ± 2.3	20.4 ± 1.4	15.4 ± 1.4	21.7 ± 3.9	57.9 ± 8.4	87.5 ± 6.6	
*P value*	0.002*		0.001*		0.002*		0.006*		0.002*		

Excellent cases (5) and good cases (7).

*p value is statistically significant.

**Figure 1:**
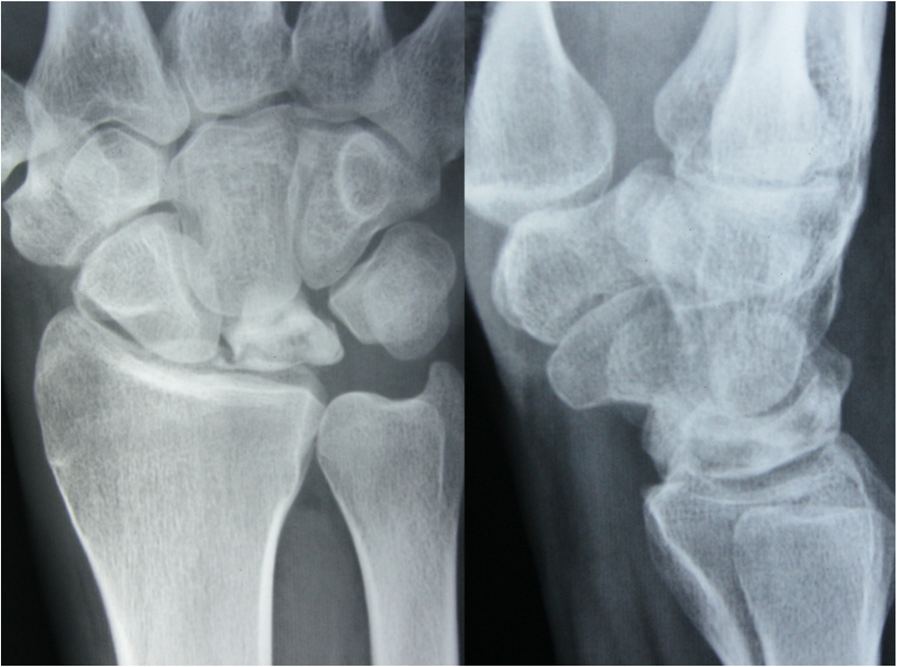
**Preoperative wrist radiographs of stage II Kienböck’s disease.**

**Figure 2:**
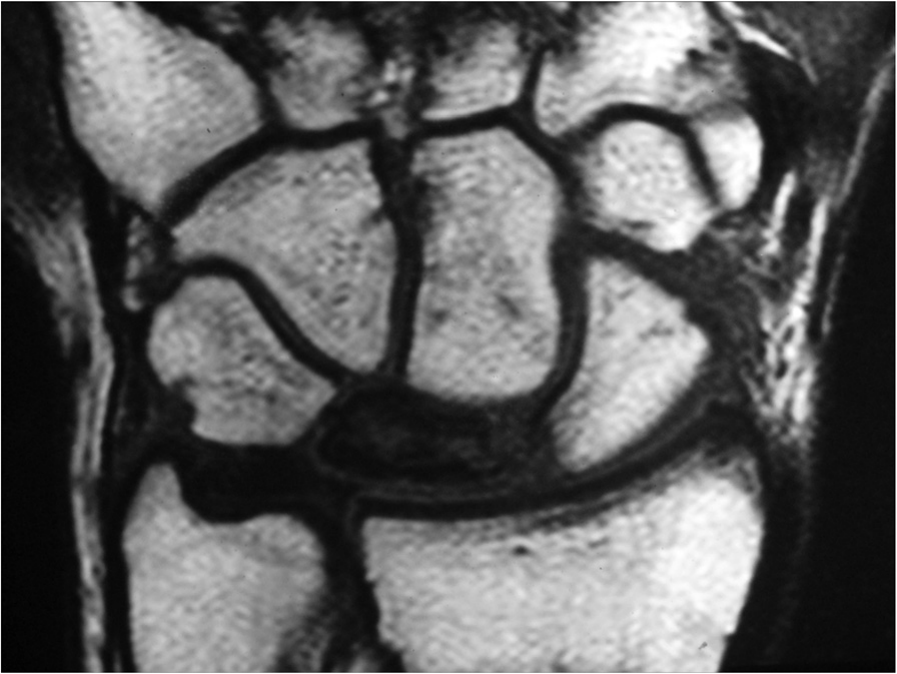
**T1-weighted MRI showing decreased signal intensity in the lunate bone.**

Both pre-operative and post-operative wrist radiographs were obtained in all patients in standard fashion (Figure 1). The post-operative radiographs were obtained immediately after surgery, after 6 weeks, and then routinely every 2 weeks until fusion healing was achieved. All patients had MRI both before surgery and at the 12th month of follow-up [8,9] (Figure 2). Radiographic staging was evaluated according to Lichtman’s methodology [10]. In addition, the scapho-capitate angle (the angle between the longitudinal axes of the capitate and scaphoid) on the postero-anterior radiograph of the wrist were evaluated both pre-operatively and 12 months post-operatively. Patients with wrist pain who were diagnosed as having Lichtman stage II or stage IIIA Kienböck’s disease with neutral ulnar variance were included in this study. Those patients with radiographic evidence of radiocarpal and/or midcarpal arthrosis (Lichtman stage IV) were excluded from the study.

All patients had uniform technique of distal capitate shortening with concurrent capitometacarpal fusion. The average follow-up period was 20.7 ± 11.2 months (range; 12–47 months). Average time of healing of the capitometacarpal fusion was observed.

### MRI examination

MRI examination was done to all patients using MR system (Philips Gyroscan Intera 1.0, Philips Medical Systems, Eindhoven, the Netherlands) with a dedicated wrist coil (Philips wrist coil, Philips Medical Systems, Eindhoven, The Netherlands). Fat-saturated T2-weighted FSE sequences (TR/TE 3,000/90, slice thickness 2 mm, interslice gap 0.2 mm) in the coronal, sagittal, and axial plane were acquired; coronal 3D gradient-echo (58/12; flip angle, 10°; matrix size, 256 × 128; number of excitations, 2; field of view, 8 cm; slice thickness, 1.2 mm; interslice gap, 0 mm) and fat saturated T1-weighted sequences (TR/TE 400/16, slice thickness 2 mm, interslice gap 0.2 mm) in the coronal plane was done without IV contrast. T2 with fat saturation sequence was the most useful sequence used to assess revascularization process.

### Image interpretation

Image interpretation was done with one musculoskeletal (MSK) radiologist of 15 years’ experience in MRI of the MSK reading, he was blinded of the clinical findings and surgical results, and the final diagnosis depended on both standard radiographs and MRI results.

### Surgical technique

**Figure 3:**
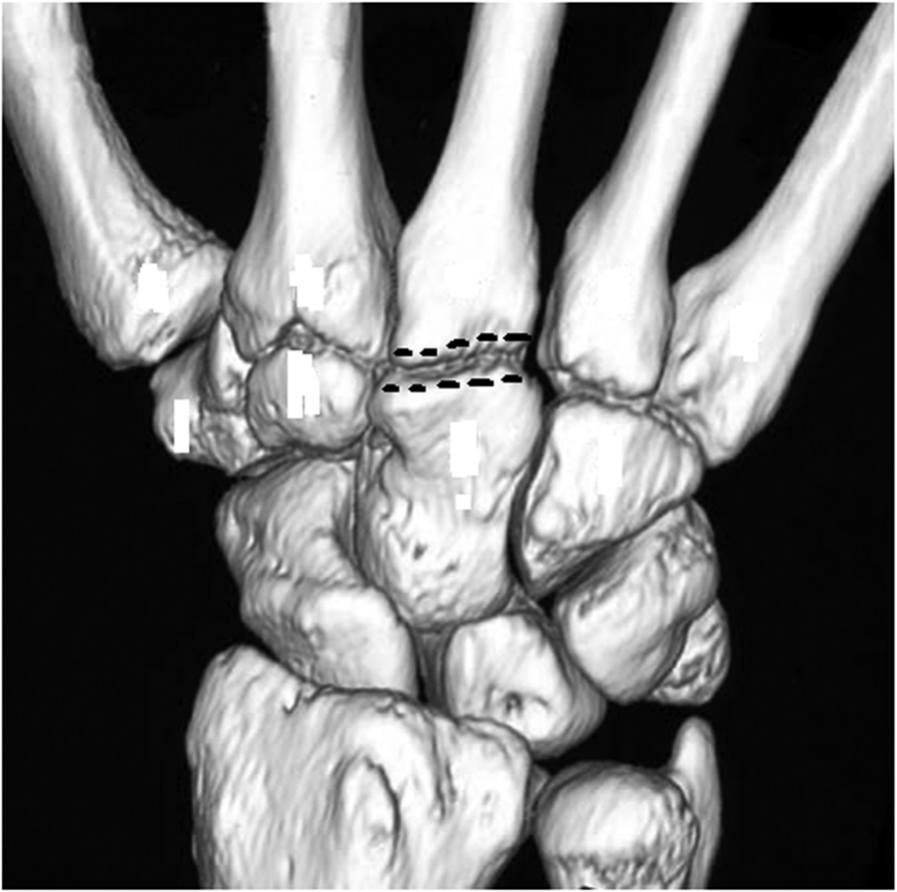
**Landmarks on the 3D CT reconstruction of the wrist denoting the site of the saw cuts.**

**Figure 4:**
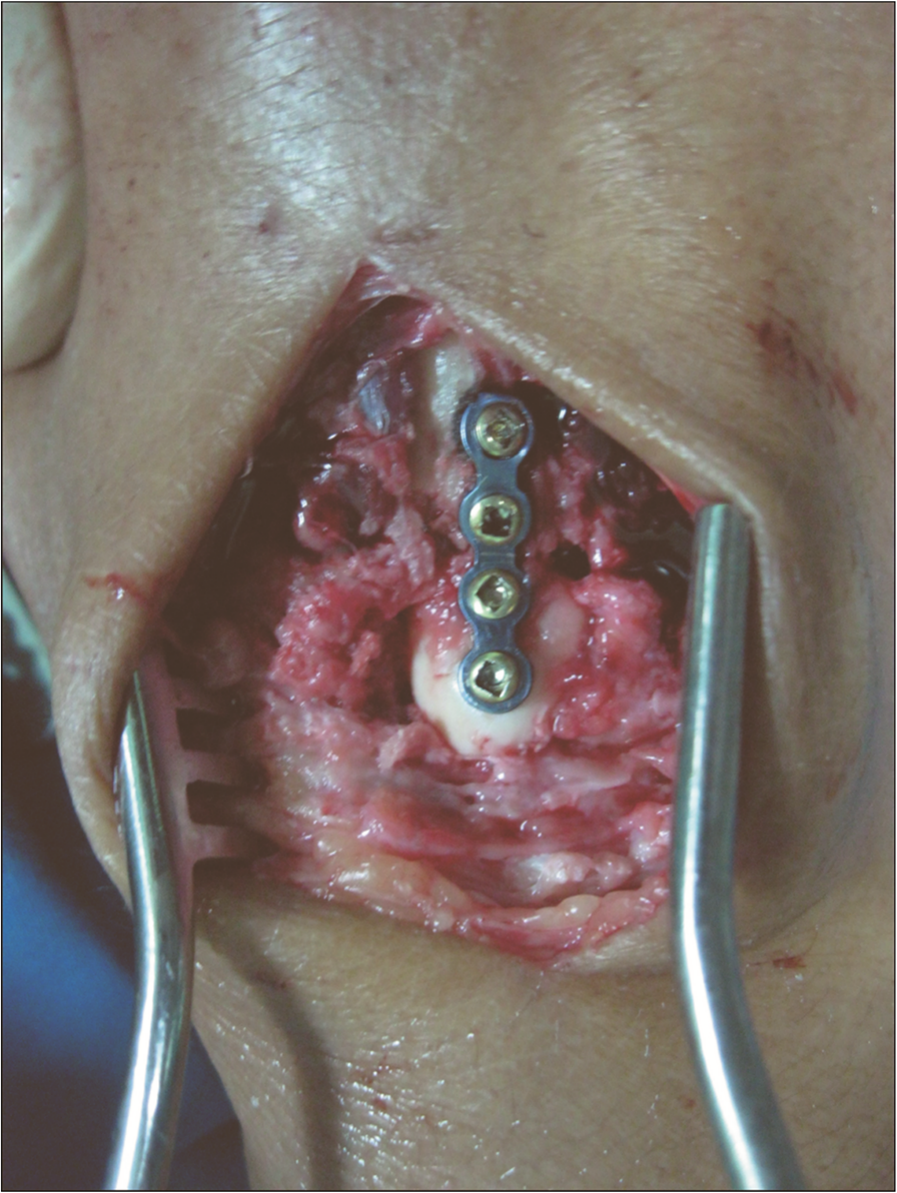
**Low profile miniplate and screw fixation of the capitometacarpal fusion.**

The surgery was performed under regional or general anesthesia with the patient in supine position and the arm on a hand operating table under tourniquet control. A 3-cm midline longitudinal dorsal incision centered over capitometacarpal joint was made, the tendons of the 4th compartment were retracted to the ulnar side, and the capsule was incised longitudinally to expose the capitometacarpal joint. The location of the capitometacarpal joint was routinely confirmed by using fluoroscopy. With a fine oscillating saw, 1.0–1.5 mm wafer of bone and cartilage was resected from the distal end of the capitate and a similar wafer was resected from the base of the 3rd metacarpal, creating a 2–3 mm defect (Figure 3). The saw cuts were completed using small osteotomes to prevent an injury to the flexor tendons on the palmar surface of the capitometacarpal joint. The bone surfaces of the capitate and the 3rd metacarpal were compressed using reduction forceps and stabilized with low profile miniplate and screws (Figure 4). The wound was closed over the approximated capsule and 4th extensor compartment, and the wrist was placed into a short arm volar splint. Limited wrist motion as tolerated was allowed at 6 weeks. Between 8 and 12 post-operative weeks, active assisted and passive motion were initiated, as well as progressive resistance exercises for hand and wrist strengthening. After solid fusion was achieved, strengthening exercises were added as the swelling and discomfort subsided.

### Statistical analysis

Statistical analysis was performed for comparisons between pre-operative and post-operative findings with the use of paired *t* tests. The pre-operative and post-operative values of the scapho-capitate angle were evaluated and correlated to the clinical parameters and the modified Mayo wrist score using the Pearson’s correlation, independent sample *T* test, and Spearman’s rho correlation. Statistical significance was determined to be present at *p* < 0.05; all data analysis was calculated with statistical software (SPSS for Windows, version 13; SPSS Inc., Chicago, IL).

## Results

Eight of 12 patients were diagnosed as having Lichtman stage II Kienböck’s disease (66.7%) and four were classified as stage IIIA (33.3%). Pre-operatively, all patients exhibited neutral ulnar variance.

**Figure 5:**
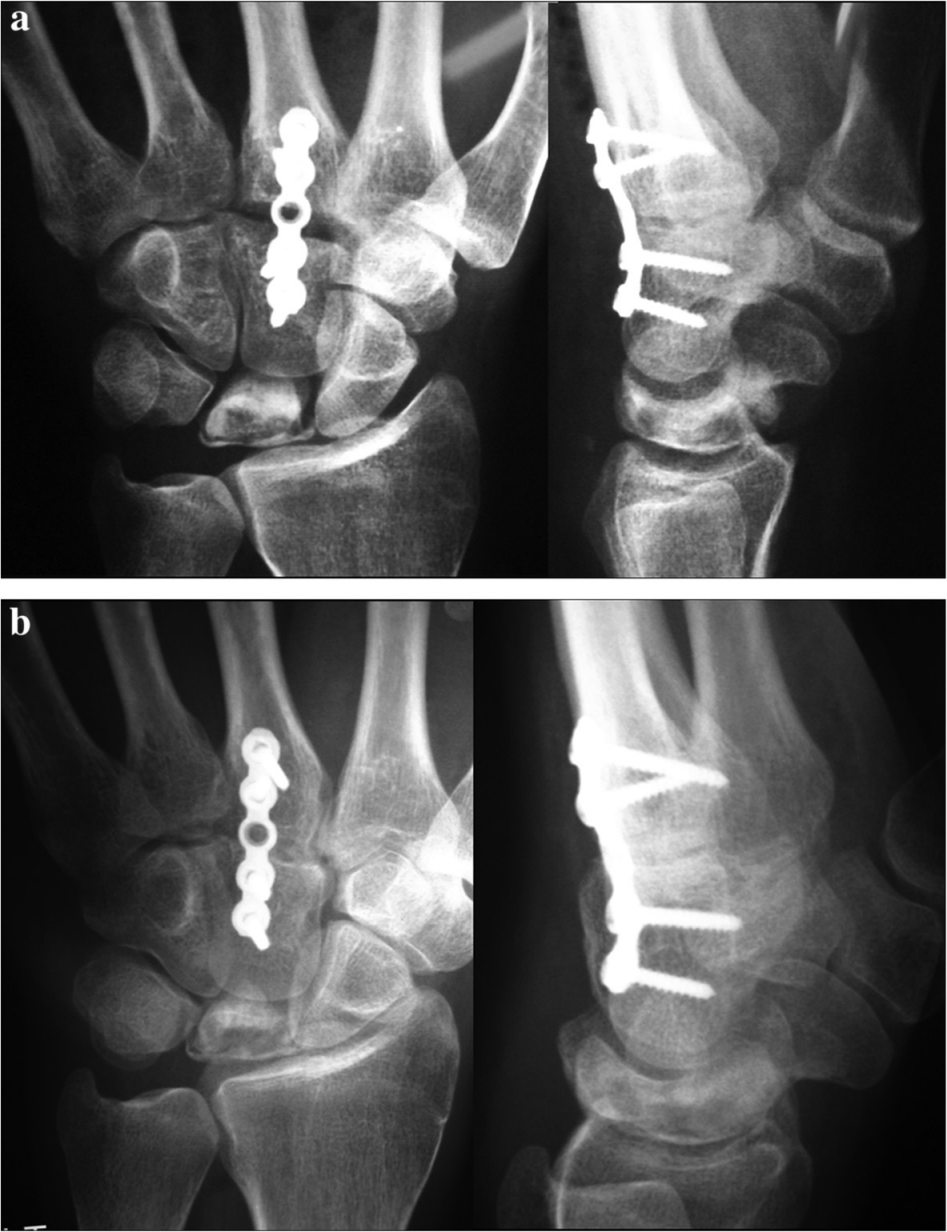
**Early and postoperative radiology. (a)** Early postoperative radiology. **(b)** Late postoperative radiology after fusion of capitometacarpal joint.

All patients achieved bony union at the fusion site within 8–16 weeks after surgery (mean: 11.5 ± 2.4 weeks) (Figure 5). Regarding wrist pain, 12 months post-operatively, all patients participating in the study achieved statistically significant differences from the pre-operative pain score (*p* = 0.002). At 12 months post-operatively, eight patients managed to return to their normal occupations and practice their daily activities in a normal pattern (66.7%) and in whole, the post-operative functional scoring attained a statistically significant level (*p* = 0.001).

Regarding total wrist arc of motion compared to the normal side, all patients achieved an arc of motion ranging from 80%–100% of the normal side (*p* = 0.002). For the differential arc of motion, the post-operative range of motion was statistically different from the pre-operative range regarding the flexion/extension and pronation/supination range (*p* = 0.001 and *p* = 0.016, respectively) but it was not significantly different regarding the ulnar/radial deviation range (*p* = 0.262) (Table 4). Similarly, grip strength compared to the normal side was 90%–100% in six patients and 80%–89% in four patients (*p* = 0.006).

**Table 4:** **Results of ROM both pre- and post-operatively**

**Patient**	**F/E AOM (pre-operatively)**	**F/E AOM (post-operatively)**	**P/S AOM (pre-operatively)**	**P/S AOM (post-operatively)**	**U/R deviation AOM (pre-operatively)**	**U/R deviation AOM (post-operatively)**
*1*	100	115	150	160	50	52
*2*	85	120	120	148	30	40
*3*	80	110	80	150	25	40
*4*	90	100	70	140	30	35
*5*	70	110	90	140	35	40
*6*	87	100	135	140	48	45
*7*	112	120	155	155	42	40
*8*	107	100	160	160	40	35
*9*	80	100	162	165	60	50
*10*	85	115	120	160	40	50
*11*	88	98	156	155	48	50
*12*	102	110	160	160	62	62
*Mean ± SD*	90.5 ± 12.4	108.2 ± 8.3	129.8 ± 33.6	152.8 ± 9	42.5 ± 11.6	44.9 ± 8.1
*P value*	0.001*		0.016*		0.262	

*p value is statistically significant.

The final post-operative modified Mayo wrist score ranged from 80–100 points (mean: 87.5 ± 6.6 points) which was statistically significant from the pre-operative score (*p* = 0.002) (Table 3). According to the post-operative grading; five cases rated excellent and seven cases rated good while no case rated either satisfactory or poor (Table 3). The previous results confirm the obvious improvement regarding the grading and scoring after this new technique.

The mean pre-operative scapho-capitate angle was 29.75 ± 3.44 while the mean post-operative value was 33.67 ± 4.77 with a statistically significant difference (*p* < 0.001) (Table 5). The pre-operative scapho-capitate angle values were positively correlated to the following parameters; post-operative pain, ulnar/radial deviation, and final score (*p* = 0.001, 0.027, 0.021, respectively). The post-operative scapho-capitate angle values were positively correlated to the same parameters (*p* = 0.001, 0.004, 0.002, respectively). Other parameters had no correlation to this angle (Table 6).

**Table 5:** **Scapho-capitate angle values, means, SD, and** ***p*** **value using paired sample** ***T*** **test**

**Patient (N)**	**Pre-operative scapho-capitate angle (degrees)**	**Post-operative scapho-capitate angle (degrees)**
1	32	38
2	28	32
3	30	33
4	26	28
5	28	30
6	34	39
7	29	31
8	24	26
9	27	33
10	36	42
11	31	34
12	32	38
*Mean ± SD*	29.75 ± 3.44	33.67 ± 4.77

*p* < 0.001.

**Table 6:** **Correlation of the scapho-capitate angle to the assessed clinical parameters both pre-operatively and post-operatively using Pearson’s correlation**

	**Pre-operative scapho-capitate angle**		**Post-operative scapho-capitate angle**	
	**R**	**P**	**R**	**P**
*Post-operative scapho-capitate angle*	*0.957*	<0.001*		
*Pre-operative F/E AOM*	−0.091	0.779	−0.088	0.786
*Post-operative F/E AOM*	0.263	0.408	0.236	0.460
*Pre-operative P/S AOM*	0.098	0.762	0.219	0.494
*Post-operative P/S AOM*	0.080	0.805	0.244	0.445
*U/R deviation AOM (preoperatively)*	0.271	0.394	0.437	0.156
*U/R deviation AOM (postoperatively)*	*0.633*	0.027*	*0.759*	0.004*
*Pre-operative pain*	0.307	0.332	0.421	0.173
*Post-operative pain*	*0.834*	0.001*	*0.838*	0.001*
*Pre-operative functional status*	-------	--------	--------	--------
*Post-operative functional status*	0.161	0.617	0.180	0.575
*Pre-operative range of motion*	0.219	0.494	0.253	0.428
*Post-operative range of motion*	0.206	0.521	0.286	0.368
*Pre-operative grip strength*	−0.069	0.832	−0.176	0.584
*Post-operative grip strength*	0.373	0.232	0.571	0.053
*Pre-operative total score*	0.295	0.351	0.379	0.225
*Post-operative total score*	*0.653*	0.021*	*0.797*	0.002*

*p value is statistically significant.

Both pre-operative and post-operative scapho-capitate angle values were significantly correlated to the grading of the modified Mayo wrist scoring (*p* = 0.002 and 0.029, respectively) (Table 7). In addition the degree of both pre-operative and post-operative scapho-capitate angle, values were positively correlated to scoring system which meant that the larger the angle, the better the score (*p* = 0.034 and 0.002, respectively) (Table 8).

**Table 7:** **The relation of the scapho-capitate angle and the modified Mayo wrist score using independent sample** ***T*** **test**

	**Modified Mayo wrist score**		***P*** **value**
	**Good**	**Excellent**	
*Scapho-capitate angle (pre-operative)*	30.57 ± 2.82	38 ± 3.24	0.002*
*Scapho-capitate angle (post-operative)*	28 ± 2.38	32.2 ± 3.35	0.029*

*p value is statistically significant.

**Table 8:** **Correlation of the scapho-capitate angle to the modified Mayo wrist scoring using Spearman’s rho correlation**

	**Pre-operative scapho-capitate angle**		**Post-operative scapho-capitate angle**	
	**R**	**P**	**R**	**P**
*Modified Mayo wrist score*	0.614	0.034*	0.786	0.002*

*p value is statistically significant.

**Figure 6:**
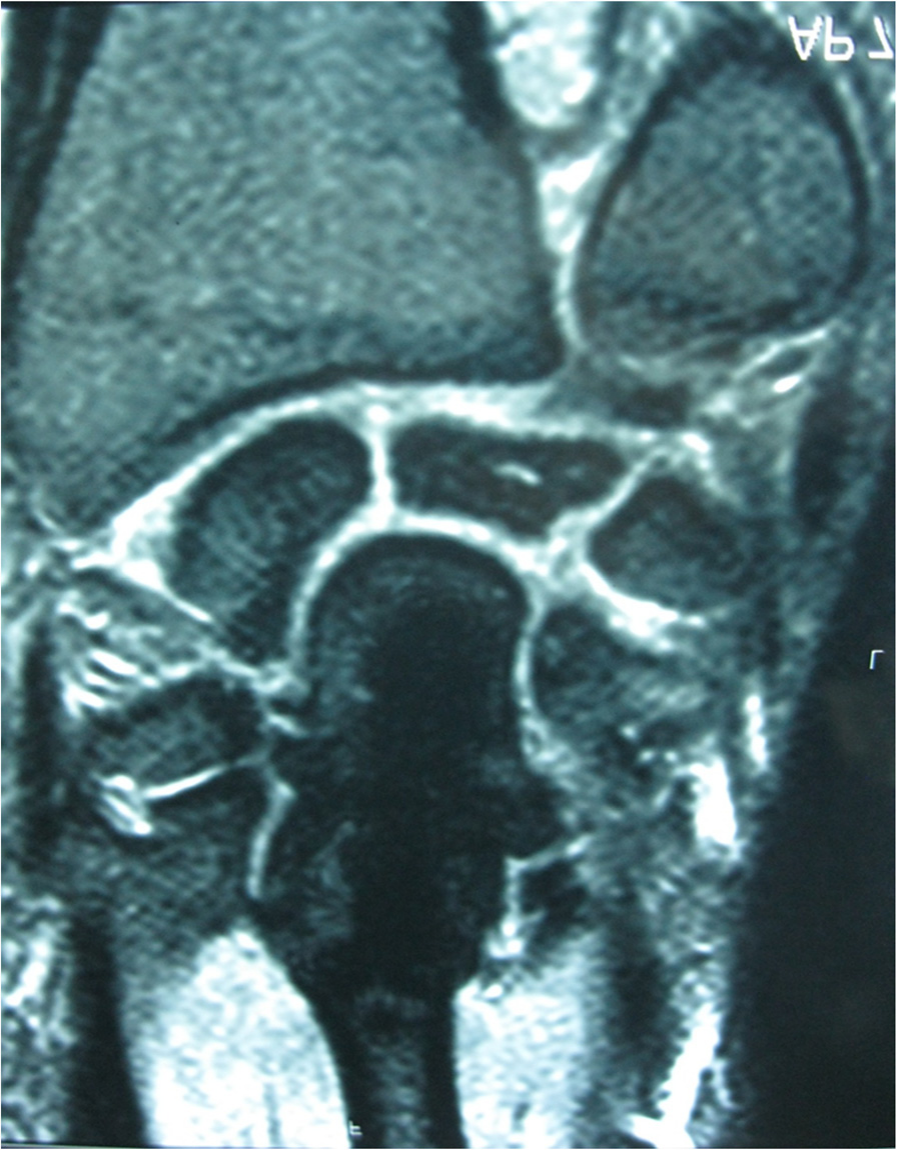
**Final follow-up MRI showing better lunate revascularization.**

MRI examination at 12 months follow-up demonstrated both solid union of the fusion in all cases and better lunate revascularization in four patients; one of them was diagnosed as having Lichtman stage IIIA Kienböck’s disease with neutral ulnar variance (Figure 6). There were no patient-reported complications at the end of follow-up.

## Discussion

The etiology of Kienböck’s disease remains poorly understood and that is why there is still little evidence to support any particular form of operative treatment or to indicate its superiority over conservative measures [11]. The rationale behind the standard CH fusion is to prevent secondary proximal migration of the capitate which is the same rationale behind the capitometacarpal fusion in our study; however, CH fusion alone does not reduce load transmission through the lunate [12]. In cases where ulnar variance is neutral, classic interventions such as radial shortening osteotomy can modify the wrist biomechanics and can lead to an increased risk of ulnar wrist pain [12-15].

In a recent anatomical study, significant load reduction on the lunate was evident in all specimens after capitate shortening in cases of neutral ulnar variance [2]. An average decrease of 49% was seen under a 9.8 N load and 56% under a 19.6 N load. The load was transferred to the radial and ulnar columns of intercarpal joints. In addition, more relief of pressure on the lunate after isolated capitate shortening was achieved with a shallow angle between the scaphoid and capitate in the postero-anterior radiograph. The authors of this study believe that isolated capitate shortening results in an increase in the scapho-capitate angle as this angle is directly related to the shape of the capitate with subsequent reduction of the radiolunate pressure. The mean scapho-capitate angle was reported to be 25 ± 11 in the postero-anterior radiograph in one study [16]. In another study the mean angle was 28 ± 12 (range; 7°–46°) [2]. In our study, the scapho-capitate angle was evaluated both pre and post-operatively. The mean pre-operative scapho-capitate angle was 29.75 ± 3.44 while the mean post-operative value was 33.67 ± 4.77 with a statistically significant difference (*p* < 0.001). The pre-operative scapho-capitate angle values were positively correlated to the following parameters: post-operative pain, ulnar/radial deviation, and final score (*p* = 0.001, 0.027, 0.021, respectively). The post-operative scapho-capitate angle values were positively correlated to the same parameters (*p* = 0.001, 0.004, 0.002, respectively). Both pre-operative and post-operative scapho-capitate angle values were significantly correlated to the grading of the modified Mayo wrist scoring (*p* = 0.002 and 0.029, respectively). In addition, the degree of both pre-operative and post-operative scapho-capitate angle values were positively correlated to the same scoring system which means that the larger the angle, the better the score (*p* = 0.034 and 0.002, respectively).

In another previous study, biomechanical effects of CSCHF have been studied and the authors concluded that CSCHF increased radioscaphoid mean pressure and decreased radiolunate mean pressure and had little effect on radiocarpal mean pressure [4]. In another study, after capitate shortening, the radioscaphoid joint mean pressure was significantly increased by an average of 39%, the radiolunate joint mean pressure was significantly decreased by an average of 53%, and the ulnocarpal joint mean pressure was unchanged [17]. Cumulatively, partial or complete capitate shortening is believed by many authors to decrease force on the lunate by transferring the axial load onto the scaphoid, radius, and triquetrum [1]. Based on these biomechanical analyses, we believe that reduction in the radiolunate load is maintained by both capitate shortening and capitometacarpal fusion. In addition, the ulnar and lateral pillars of the carpus are hypothetically loaded equally. This was the basis of our hypothesis.

In this study, our procedure combined capitate shortening with capitometacarpal fusion for the treatment of Lichtman stages II and IIIA Kienböck’s disease in neutral ulnar wrists. We planned to shorten the capitate from its distal end together with fusion of the capitometacarpal joint. The authors emphasized a direct approach to the capitometacarpal joint without radiocarpal joint opening with a threefold advantage of respecting the radiocarpal articulation, limiting its consecutive stiffness, and preventing more devascularization of the lunate. Fixation of the capitometacarpal fusion was maintained by low profile 2.0 mm miniplate and screws, and capitohamate fusion was not performed. The fixation hardware was not removed as it did not cause any complication and it did not interfere with later on radiographic or MRI examination. A series of 12 patients were operated on, with a mean follow-up of 20.7 ± 11.2 months. Clinically, the patients were improved regarding the degree of pain, grip strength, total and differential arc of motion, return to normal daily activities, and the final modified Mayo wrist score. All post-operative parameters were statistically significant with the exception of the ulnar/radial arc of motion (*p* = 0.262).

We believe that our procedure obviated the need for capitohamate fusion (limited intercarpal arthrodesis) which in turn minimized the possibility of later osteoarthritic changes which were noted by some authors [18]. In addition, the distal fusion of the capitate overcame the problem of endangered vascularity of its proximal pole. The post-operative grip strength was comparable and even better than the results of previous studies [19-21] and the results regarding pain and force were better than in other studies of radial shortening osteotomy [22,23]. In our series, no non-union was observed nor was there any proximal capitate necrosis. The healing time in our series was comparable to previous studies provided that the method of fixation was universal in all our patients [24]. Waitayawinyu et al. [24] reporting a series of 14 patients who underwent capitate shortening osteotomy (with or without hamate shortening) associated with a 3rd metacarpal-based vascularized bone graft yielded comparable results in terms of strength improvement and post-operative range of motion.

MRI examination at 12 months follow-up demonstrated both solid union of the fusion in all cases and better lunate revascularization in four patients; one of them was diagnosed as having Lichtman stage IIIA Kienböck’s disease with neutral ulnar variance. This study lacks the sequential and long term MRI examination that may demonstrate better revascularization of the lunate.

## Conclusions

In summary, our results suggest that distal capitate shortening with capitometacarpal fusion is an effective and reliable procedure to achieve satisfactory pain relief, arc of motion, and improved grip strength in the early stages of Kienböck’s disease, with neutral ulnar variance, without increased risk of proximal capitate necrosis or secondary proximal migration of the capitate. It allows better lunate revascularization and may accelerate it. We believe that a biomechanical study should be done to verify our results and foresee the possible consequences that may take place in the middle carpometacarpal column and other intercarpal joints particularly after the significant increase in the scapho-capitate angle values.

## Abbreviations

CSCHF: capitate shortening with capitohamate fusion
MSK: musculoskeletal
